# Empagliflozin’s cardioenergetic protective effects through PPARα pathway modulation in heart failure

**DOI:** 10.3389/fphar.2025.1636810

**Published:** 2025-10-17

**Authors:** Hua Wei, Menghua Yin, Junshun Chang, Bo Feng, Qin Zhou, Xirong Li, Ping Wu, Xiaoshan Guo, Siyuan Chen, Bao Li, Sijin Li

**Affiliations:** ^1^ Department of Nuclear Medicine, First Hospital of Shanxi Medical University, Taiyuan, Shanxi, China; ^2^ Post-doctoral workstation, Second Hospital of Shanxi Medical University, Taiyuan, Shanxi, China; ^3^ Collaborative Innovation Center for Molecular Imaging of Precision Medicine, Shanxi Medical University, Taiyuan, Shanxi, China

**Keywords:** empagliflozin, PPARα signaling pathway, heart failure, ^18^F-FDG PET/CT imaging, myocardial metabolism

## Abstract

**Background:**

Heart failure (HF) pathology is complex and seriously life-threatening. SGLT2 inhibitors, as one of the new quadruple drugs for HF treatment, have a complex mechanism for improving HF. Energy metabolism is one of the important aspects of HF pathology, and the PPARα signaling pathway plays an important role in energy metabolism. Therefore, this study aims to observe changes in the PPARα signal transduction pathway in chronic HF by ^18^F-FDG MicroPET/CT imaging. Based on the myocardial metabolic imaging of ^18^F-FDG MicroPET/CT, this study aims to verify the mechanism of SGLT2 inhibitor treatment in rats with HF through the PPARα signal transduction pathway of energy metabolism and provide an imaging diagnostic basis.

**Results:**

In ^18^F-FDG PET/CT myocardial metabolic imaging, pretreatment myocardial glucose metabolism rate (MRGlu) levels in the HC group of HF rats were significantly higher than that in the other three groups. Post-treatment, MRGlu and glucose uptake decreased markedly in the empagliflozin (EMPG) group, while no significant changes were observed in the fenofibrate (FF) group. Compared with normal healthy rats, HF model rats showed a significant increase in MRGlu, and the expression of the lipid metabolism pathway proteins (PPARα, RXRα, CPT1α, and CD36) and the energy metabolism pathway proteins (AMPKα and sirt1) were significantly inhibited, while the expression of the glycolytic pathway protein (GLUT4) was enhanced. After 4 weeks of drug treatment in HF model rats, EMPG showed the same lipid metabolism pathway proteins (PPARα, RXRα, and CPT1α) and energy metabolism pathway proteins (AMPKα and sirt1) as FF, but only EMPG showed a significant decrease in MRGlu, inhibition of glycolytic pathway protein (GLUT 4) expression, and decreased cardiac fibrosis in HF rats.

**Conclusion:**

This study led to the following conclusions. 1) Rats with HF showed a significant increase in MRGlu compared with healthy rats. 2) Empagliflozin can improve the energy supply efficiency of the heart in rats with chronic HF by inhibiting glucose metabolism and promoting lipid metabolism, thereby ameliorating energy metabolism in chronic HF. 3) ^18^F-FDG MicroPET/CT can observe the energy metabolism changes of HF, and the MRGlu can provide quantitative data for the changes of HF energy metabolism.

## 1 Background

Heart failure (HF) represents a complex clinical syndrome characterized by profound structural and functional cardiac abnormalities, resulting in elevated intra-cardiac pressures and/or inadequate cardiac output. This condition manifests through a spectrum of characteristic symptoms and signs, presenting either at rest or during exertion, and is associated with substantial morbidity and mortality worldwide. Currently, HF affects over 64 million people worldwide (Savarese et al., 2023; Das et al., 2021). The structural and functional changes in the heart ultimately result in HF, involving mechanisms such as alterations in energy metabolism (Conte et al., 2023) (Hundertmark et al., 2023; Da Dalt et al., 2023), autophagy, apoptosis, oxidative stress, mitochondrial dysfunction, angiogenesis, and dysregulation of signaling pathways (Chen et al., 2024). Energy metabolism dysfunction is a critical component in the pathogenesis of HF. To maintain cardiac function, cardiomyocytes must continuously produce large amounts of adenosine triphosphate (ATP). Under normal physiological conditions, ATP production primarily relies on fatty acid oxidation (60%–90%), followed by glucose oxidation (10%–30%), with ketone bodies contributing approximately 5% of the total ATP (Saucedo-Orozco et al., 2022). In the early stages of HF, the expression of fatty acid oxidation enzymes (FAO) is downregulated, which shifts myocardial energy metabolism from predominantly fatty acid oxidation to anaerobic glycolysis. In advanced HF, insulin resistance progresses, leading to reduced glucose metabolism, and the myocardium oxidizes ketone bodies (particularly β-hydroxybutyrate, β-OHB), lactate, and amino acids, shifting energy metabolism toward ketone body utilization (Razeghi et al., 2001). For the myocardium, ketone bodies are a more efficient fuel than fatty acids and glucose, increasing myocardial energy efficiency by 24% and enhancing the energy supply (Marton et al., 2021). However, increased ketone body utilization can lead to the accumulation of toxic lipids such as palmitic acid, sphingolipids, and ceramides, which directly affect cell membrane integrity and promote cardiomyocyte apoptosis, further exacerbating HF (Da Dalt et al., 2023). Therefore, altering cardiac metabolic substrates to improve energy supply efficiency while reducing the accumulation of toxic substances may ameliorate energy metabolism dysregulation in HF patients.

The PPAR family, which controls various intracellular metabolic processes, plays a significant role throughout the cardiac fatty acid oxidation process (Christofides et al., 2021). PPARα, one of the three subtypes of the PPAR family, is highly expressed in cardiomyocytes. Activated by endogenous ligands such as free fatty acids (FFA), PPARα participates in every step of myocardial fatty acid oxidation phosphorylation, including fatty acid uptake via fatty acid translocase (FATP1/CD36), transport into the mitochondria via carnitine palmitoyltransferase 1 (CPT1α) on the outer mitochondrial membrane, and the entire mitochondrial β-oxidation process, making it a crucial determinant of myocardial energy production. Conversely, PPARα inactivation promotes fatty acid accumulation and suppresses the expression of target genes involved in FAO, including CPT1α (Li et al., 2018). Reduced PPARα expression has also been observed in the pathology of pressure overload-induced HF (Kaimoto et al., 2017). Therefore, regulating PPARα gene expression may alter cardiac energy metabolism in HF patients.

SGLT2 inhibitors were initially developed as novel oral hypoglycemic agents. In 2015, the EMPA-REG OUTCOME study first demonstrated that SGLT2 inhibitors could improve cardiovascular outcomes in patients with type 2 diabetes (Zinman et al., 2015). Subsequently, large-scale cohort trials such as DAPA-HF, EMPEROR-Reduced, and EMPEROR-Preserved revealed that the cardiovascular benefits of SGLT2 inhibitors are independent of glycemic control, significantly reducing the risk of hospitalization for HF, out-of-hospital worsening, and improving prognosis (Zannad et al., 2020; Packer et al., 2021) (Anker et al., 2021). Consequently, in 2021, SGLT2 inhibitors were officially included in the “quadruple therapy” for HF treatment guidelines. However, numerous and complex mechanisms exist by which SGLT2 inhibitors improve HF(Pabel et al., 2021; Salvatore et al., 2022), and the specific pathways remain unclear. Recent studies have shown that SGLT2 inhibitors can regulate PPARα expression, improve mitochondrial function, and promote fatty acid oxidation in *in vivo* and *in vitro* experiments (Wei et al., 2020). Based on the observation that HF patients exhibit varying degrees of skeletal muscle disorders, scholar Lv Jiayu suggested that mitochondrial abnormalities play a key role. Research has found that SGLT2 inhibitors can modulate intracellular signaling to adjust oxidative metabolism and reduce oxidative stress damage, including the PPARα signaling pathway (Lv et al., 2022). Zhang Weiwei investigated the effects of the SGLT2 inhibitor dapagliflozin on cellular senescence in atherosclerotic mice and found that it may exert its effects through direct interaction with the RXRα protein (Zhang et al., 2024). Given that RXRα is a ligand for PPARα, it is plausible that dapagliflozin also affects PPARα expression. Moreover, in cardiovascular research, PGC-1α (PPARγ coactivator-1α) agonists and SGLT2 inhibitors have shown similar effects in reducing cardiovascular events (Zelniker et al., 2019). Considering the crucial role of PPARα in myocardial energy production, it is reasonable to hypothesize that the beneficial effects of SGLT2 inhibitors on HF patients may be mediated through the regulation of the PPARα signaling pathway to enhance cardiac energy supply, thereby improving HF.

Therefore, this study aims to explore the role of SGLT2 inhibitors in the PPARα signaling pathway from the perspective of myocardial energy metabolism. Previous studies have measured myocardial phosphocreatine and ATP (Hundertmark et al., 2023) or used hyperinsulinemic–euglycemic clamp methods (Succurro et al., 2020) to calculate myocardial glucose metabolic rates. In this study, we will observe the distribution of ^18^F-FDG to reflect the uptake and phosphorylation of glucose *in vivo*. We will use ^18^F-FDG PET/CT myocardial metabolic imaging to observe changes in cardiac energy metabolism after SGLT2 inhibitor administration and further validate the therapeutic mechanisms of SGLT2 inhibitors in HF through pathological sections, Western blot (WB), and PCR assays.

## 2 Materials and methods

### 2.1 Rat HF model and grouping

**FIGURE 1:**
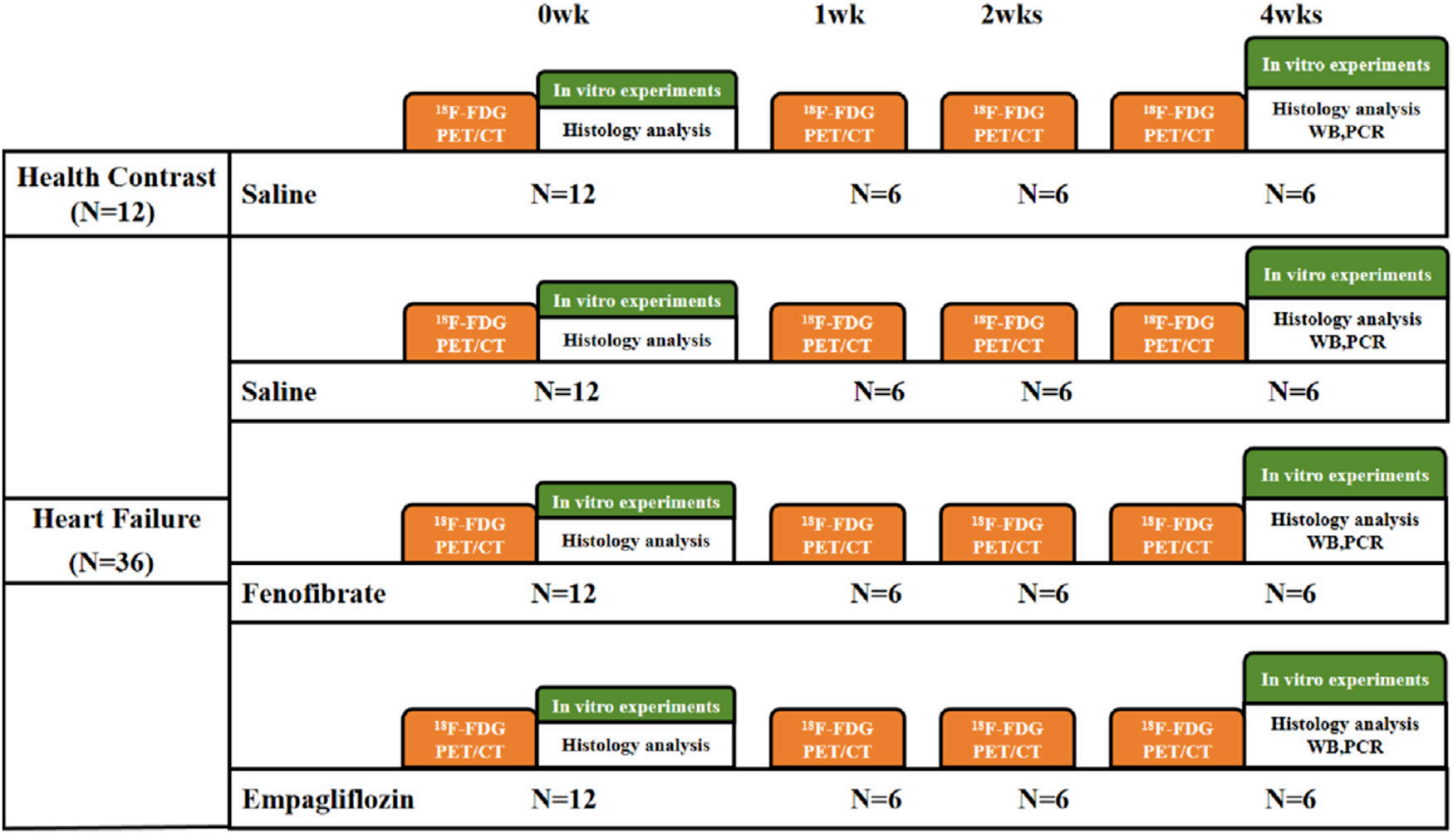
Flow chart of the experimental procedure. ^18^F-FDG PET/CT, ^18^F-FDG positron emission tomography/computed tomography; saline, 0.9% saline; WB, Western blot; PCR, polymerase chain reaction.

A total of 72 adult male SD rats (8 weeks of age, 200 g–220 g) obtained from Beijing HFK Biological Sciences Co., Ltd. (Beijing, China) were kept in adaptive housing in the laboratory for a week before the drug was administered. Mice were maintained in a pathogen-free environment (4–5 mice per cage) that was controlled at room temperature (23 °C ± 2 °C) and humidity (50% ± 5%) and had a 12-h light/dark cycle; the mice were provided with natural poplar bedding material and had *ad libitum* access to food and water, with a normal diet. After intraperitoneal injection of isoproterenol (ISO) 10 mg/kg for 4 weeks, the HF model was evaluated with ultrasound LVEF <65% (Feng and Li, 2010; Wang et al., 2016; Pan et al., 2024). A total of 36 rats in the HF model group were selected and divided into three groups: empagliflozin (EMPG 10 mg/kg, gavage, n = 12) as the SGLT2 inhibitor, fenofibrate (FF 100 mg/kg, gavage, n = 12) as the PPARα agonist group, and normal saline with the same volume (saline 0.9%, gavage, n = 12), and healthy rats of the same age were used as the healthy control group (same volume saline, gavage, n = 12). The protocol is shown in Figure 1.

### 2.2 ^18^F-FDG MicroPET/CT

#### 2.2.1 Operation

PET/CT imaging was performed using a MicroPET/CT scanner (Inveon PET/CT, Siemens, Germany). Rats were placed in a chamber connected to the isofluorinated uranium anesthesia unit, and anesthesia was induced using an air rate of 2.0 L/min containing 4.0% isofluoruric acid. The animals were then immediately placed in the prone position on the scanning bed, and the ventilation rate was reduced to 0.8 L/min–1.5 L/min with 3.0% IS. Approximately 74 MBq ^18^F-FDG was injected through the tail vein immediately using the list mode, starting continuous dynamic image acquisition for 60 min, measuring net radioactivity counts injected into the rat, and completing time decay correction. During the process of image collection, close attention was paid to the changes in respiration, heart rate, and electrocardiogram, and the anesthetic concentration of the rats was adjusted in time to stabilize the state of the rats. When it was invalid, the collection was terminated immediately, and the rats were rescued and nursed.

#### 2.2.2 Image processing

The images collected by the list mode were separated and reconstructed by 0.5 s 20 frames, 5 s 108 frames, 30 s 8 frames, 60 s 5 frames, 300 s 5 frames, and 600 s 2 frames. PMOD image processing software was used to take the last frame of image reconstruction (approximately 50 min) to outline the myocardial region of interest (area of interest, ROI). The myocardial radioactivity count curve was subjected to partial volume effect correction and attenuation effect correction (the correction factor was obtained through the model) to obtain the radioactivity–time curve of the rat myocardium. The LV ventricular model of interest was delineated, and the myocardial substrate metabolism was calculated to obtain the myocardial glucose metabolism rate (MRGlu) (Feng and Wang, 1993).

### 2.3 Histopathological analysis

Cardiac histopathological changes were observed, and the characteristics and changes of the myocardial tissue structure before and after treatment of the HF model rats were evaluated. Rats were killed before and 4 weeks after treatment in the HF rat model, and their body weight was measured; then, rat heart tissue was quickly harvested, fixed using 10% formaldehyde, and made into paraffin-embedded sections. Paraffin-embedded sections were stained with hematoxylin and eosin (HE staining) to observe the general myocardial morphology, and the degree of myocardial fibrosis was observed by MASSON staining.

### 2.4 Western blot analysis

The heart tissue samples were homogenized in ice-cold RIPA lysis buffer (50 mM Tris-HCl, pH 8.0, 0.1% sodium dodecyl sulfate, 150 mM sodium chloride, 0.5% sodium deoxycholate, and 1.0% NP-40). The supernatant was separated by centrifugation at 12,000 *g* for 15 min. The protein concentration (Bio-Rad) was determined by using the Bradford method. The sample was mixed with 2X loading buffer in a 1:1 (v/v) ratio and then boiled for 5 min. Subsequently, 50 μg of protein from each sample was loaded onto a 10% denaturing polyacrylamide mini-gel. These protein bands were transferred to a methanol pre-activated polyvinylidene fluoride (PVDF) membrane with 1% bovine serum albumin (BSA) and phosphate-buffered saline (PBS) plus 0.1% Tween 20, with gentle shaking for 2 h. Subsequently, the membranes were incubated overnight at 4 °C with the corresponding protein antibody. The beads were washed three times with PBST and incubated three times for 5 min with peroxidase-labeled goat anti-mouse antibody (Goat-Anti-Mouse IgG-HRP, 1:10,000 dilution, Chengdu Zhengneng Biotechnology Co., Ltd., Chengdu, China). The immune complexes were observed with ECL exposure. Before use, the chemiluminescence instrument was pre-cooled, the membrane was placed on the dark chamber photographic platform of the instrument, the light emitting solution was fully dropped, the door was closed, and automatic exposure photographs were taken. The densities of PPARα, RXRα, GPT1α, CD36, AMPKα, Sirt1, GLUT4, and β-actin reference bands were quantified using Image J software, and the relative density of each target protein signal to β-actin was calculated.

### 2.5 Real-time quantitative amplification analysis (RT-PCR)

Total RNA was extracted from WAT or cells using TRIzol reagent (Takara, Dalian, China). Reverse transcription reactions were performed with the help of Hunan Eco Biological Engineering Co., Ltd. (Takara). Quantitative real-time PCR (qPCR) was performed using SYBR ®PremixExTaq (Takara) following the manufacturer’s instructions. The PCR product was verified by an ABI 7500 sequencer (Applied Biosystems, Foster City, CA, United States). Pre-denaturation was carried out at 95 °C for 3 min; annealing and amplification were carried out for 10 s at 95 °C and for 30 s at 60 °C, respectively, for 40 cycles. The results for each sample were normalized to the value of β-actin.

### 2.6 Statistical analysis

Data with a normal distribution are expressed as the mean ± standard deviation. The means of continuous variables were compared between two groups using the Student’s t-test with normality or the Mann–Whitney U-test without normality. Multiple groups were compared using ANOVA, followed by Tukey’s *post hoc* test for subsequent pairwise comparisons. P < 0.05 was defined as a statistically significant difference. All statistical analyses were performed using the Prism 9.0 software (GraphPad).

## 3 Results

### 3.1 Myocardial glucose metabolism rate

**FIGURE 2:**
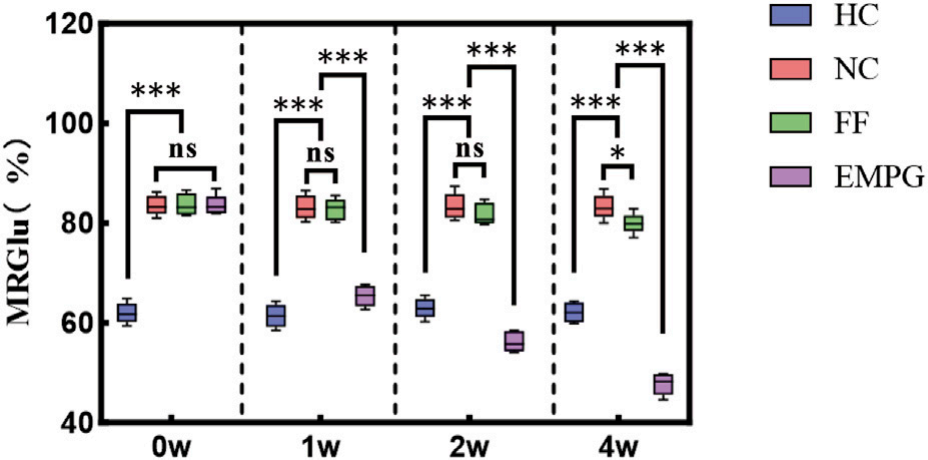
Changes in MRGlu before and after drug treatment in rats in each group. *P < 0.05, **P < 0.01, and ***P < 0.001.

Combined with Figure 2, it can be observed that at week 0, the HF model groups including the NC group, FF group, and EMPG group showed significant differences from the HC group of healthy rats, with statistically significant differences (p < 0.001). In the following 2 weeks of treatment, the MRGlu in the EMPG, NC, and FF groups was statistically significant (p < 0.001); MRGlu was not different from that in the NC group (p > 0.05). After 4 weeks of treatment, MRGlu in the EMPG group decreased statistically significantly (p < 0.001) from the FF and NC groups (p < 0.05). In conclusion, it is proved that a significant increase in glucose degradation can occur in the early stage, and after FF and EMPG treatment, the EMPG group showed a significant decrease in glycolysis; its effect was significantly stronger than that of the FF group, and the process of glycolysis can be observed through MRGlu dynamics of the nuclear medicine ^18^F-FDG PET/CT.

### 3.2 ^18^F-FDG MicroPET/CT myocardial glucose metabolism imaging

**FIGURE 3:**
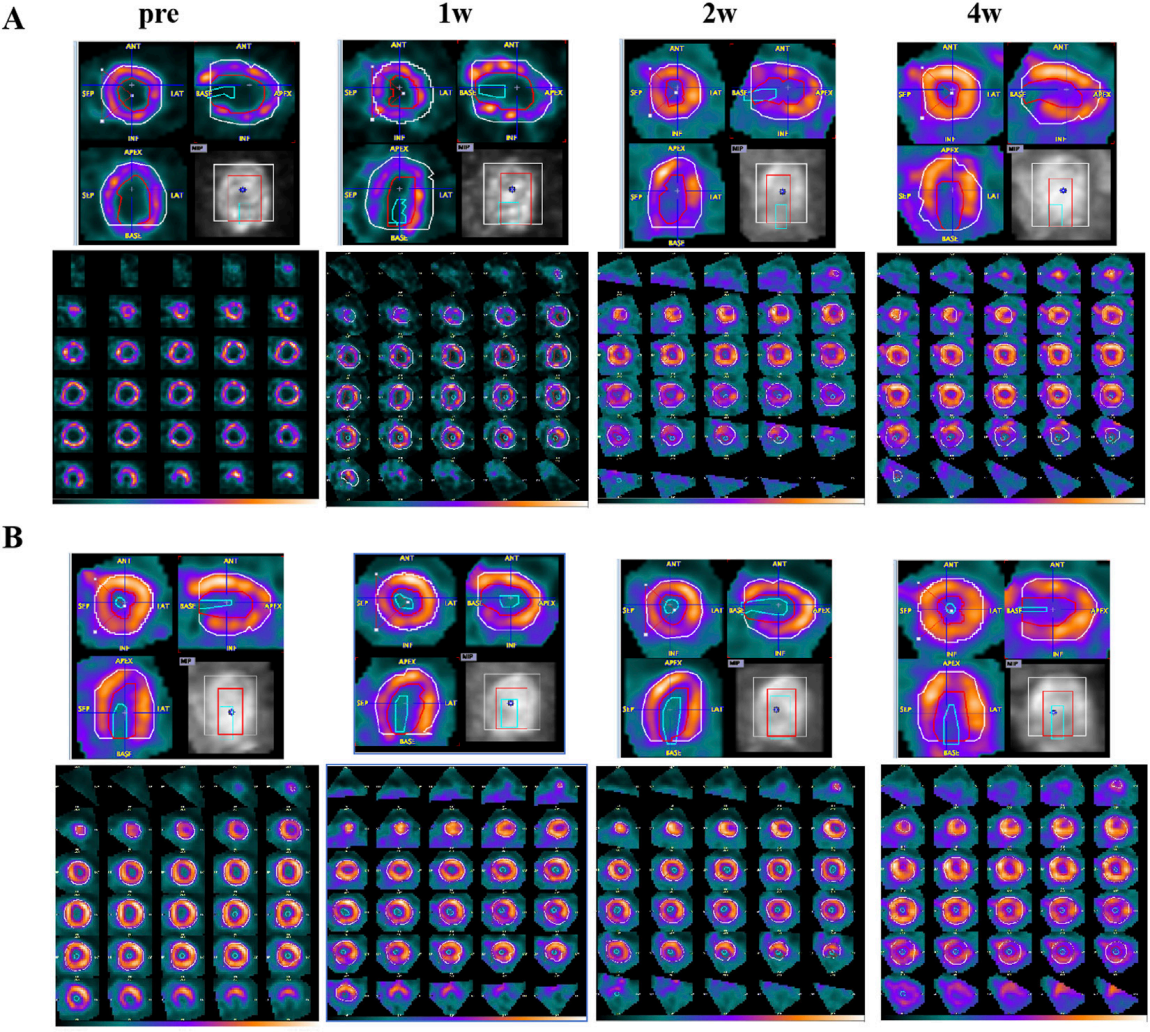
HF changes of ^18^F-FDG PET/CT images before and after 1, 2, and 4 weeks of drug treatment in rats. **(A)** FF treatment group. **(B)** EMPG treatment group.

Combined with the imaging results in Figure 3, it can be observed that the myocardial uptake was visible in the FF group, and the scattered irregular myocardial uptake defect was also visible. With the drug treatment cycle, the degree of myocardial glucose uptake was not significantly improved, and the degree of myocardial glucose uptake was not significantly changed in the scattered glucose uptake defect area (as shown in Figure 3A); in addition, in the EMPG group, it was found to gradually decrease with the time of drug treatment (as shown in Figure 3B). ^18^F-FDG MicroPET/CT myocardial glucose metabolism imaging showed a general reduction of myocardial glucose uptake in HF rats after EMPG treatment, while no significant change in myocardial glucose metabolism level in HF rats was seen after FF treatment. The changes after EMPG treatment were associated with the improvement in EMPG’s insulin resistance, which improved whole-body insulin sensitivity, thus improving peripheral glucose metabolism, reducing blood glucose levels, and prompting the heart of HF rats to use ketone body as the metabolic substrate, thus reducing cardiac glucose metabolism and improving lipid metabolism in HF rats.

### 3.3 Histopathology

**FIGURE 4:**
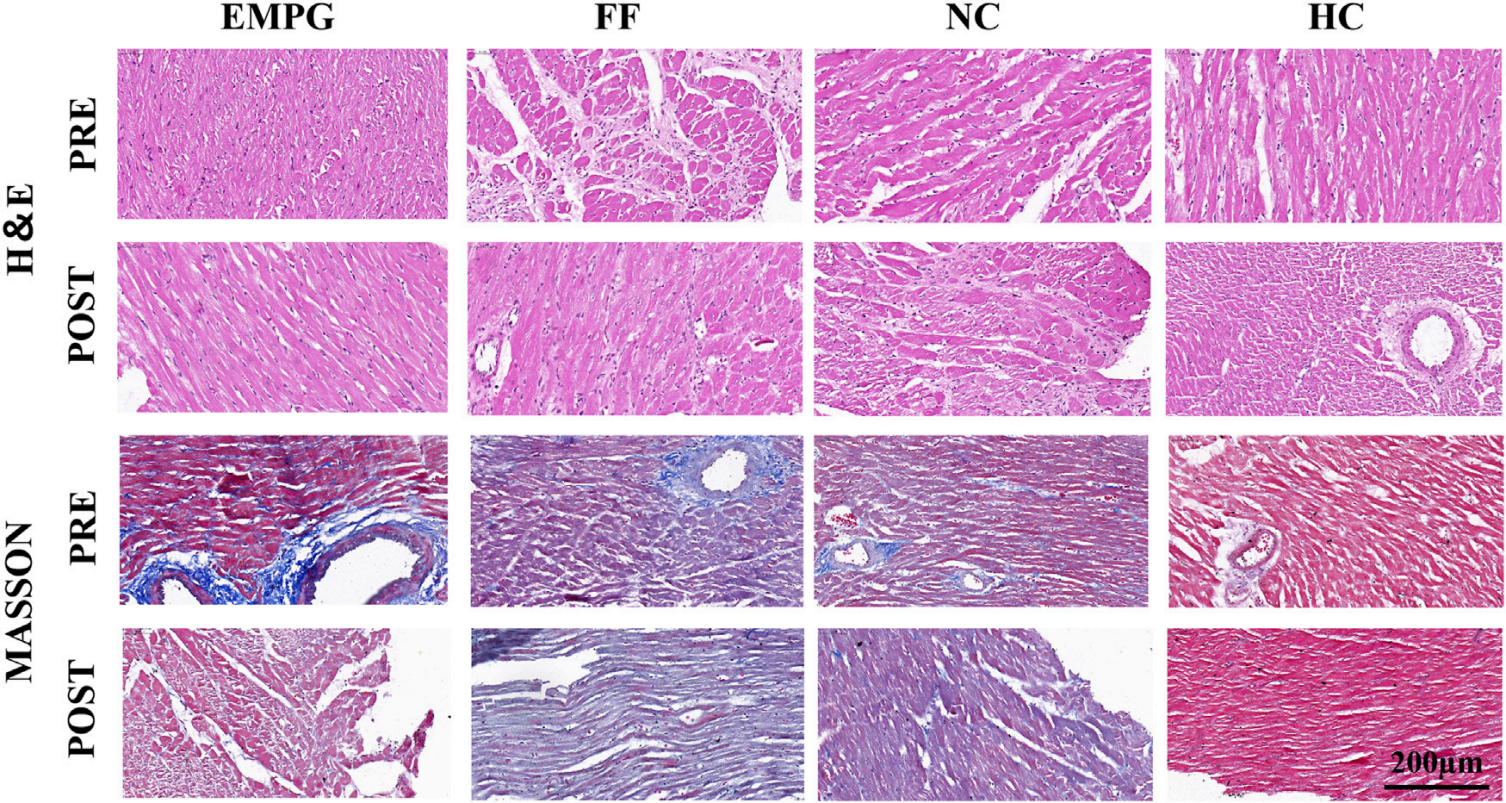
Changes in H&E staining and MASSON staining in heart sections of rats in each group (scale bar = 200 μm).

According to Figure 4, the ventricular myocardial structure of normal healthy rats (HC) was basically normal; HF rats showed obvious fibrosis and disturbance of myocardial fibers; decreased myocardial fibrosis and disturbance of myocardial fibers were seen in the EMPG group, but no significant changes were observed in the FF and NC groups. In conclusion, some recovery of ventricular remodeling was seen after EMPG treatment, while no significant changes were observed in the FF group, showing the effect of EMPG in reversing ventricular remodeling to some extent.

### 3.4 Glucose and lipid metabolism pathway proteins

**FIGURE 5:**
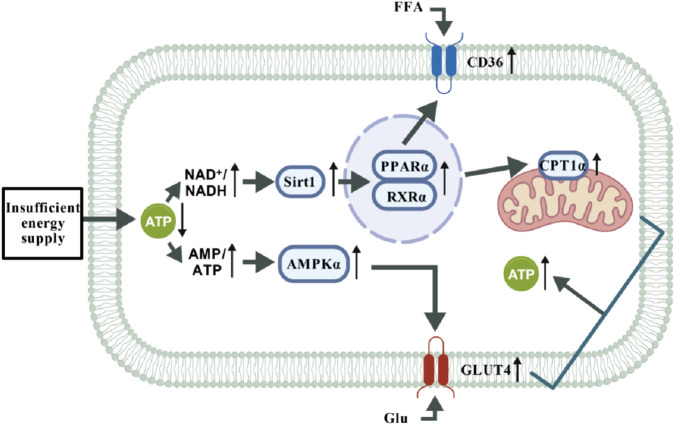
Energy supply pathways in normal cardiomyocytes under stimulation. Under conditions of energy stress (e.g., hunger or increased energy demand), elevated AMP/ATP and NAD+/NADH ratios coordinately activate the AMPK and Sirt1 signaling axis. This pathway mediates metabolic reprogramming through two major branches: Fatty acid metabolic pathway: Sirt1 deacetylates and enhances the transcriptional activity of PPARα. PPARα forms a functional heterodimer with RXRα, upregulating gene expression of the fatty acid transporter CD36 and the rate-limiting β-oxidation enzyme CPT1a, synergistically promoting fatty acid uptake and oxidation to drive ATP production. Glucose utilization pathway: AMPK directly stimulates the expression and membrane translocation of the glucose transporter GLUT4, enhancing glucose uptake and utilization to accelerate glycolysis for rapid energy supply.

In the normal heart (Figure 5), when external environmental factors impair cardiac energy supply, leading to insufficient energy provision in cardiomyocytes and a substantial reduction in ATP levels, the decreases in NAD+/NADH and AMP/ATP ratios activate Sirt1 and AMPKα, respectively. Specifically, Sirt1 activates PPARα, facilitating its dimerization with RXRα. This complex subsequently upregulates the expressions of CD36 and CPT1α proteins, which translocate to the plasma membrane and mitochondria, respectively, thereby enhancing the uptake and oxidation of free fatty acids (FFA) for energy production. Meanwhile, AMPKα stimulates the expression of GLUT4 protein, thus promoting glucose uptake and its subsequent oxidation for energy generation. Together, these two metabolic pathways coordinately increase ATP production to meet the energy demands of the organism.

**FIGURE 6:**
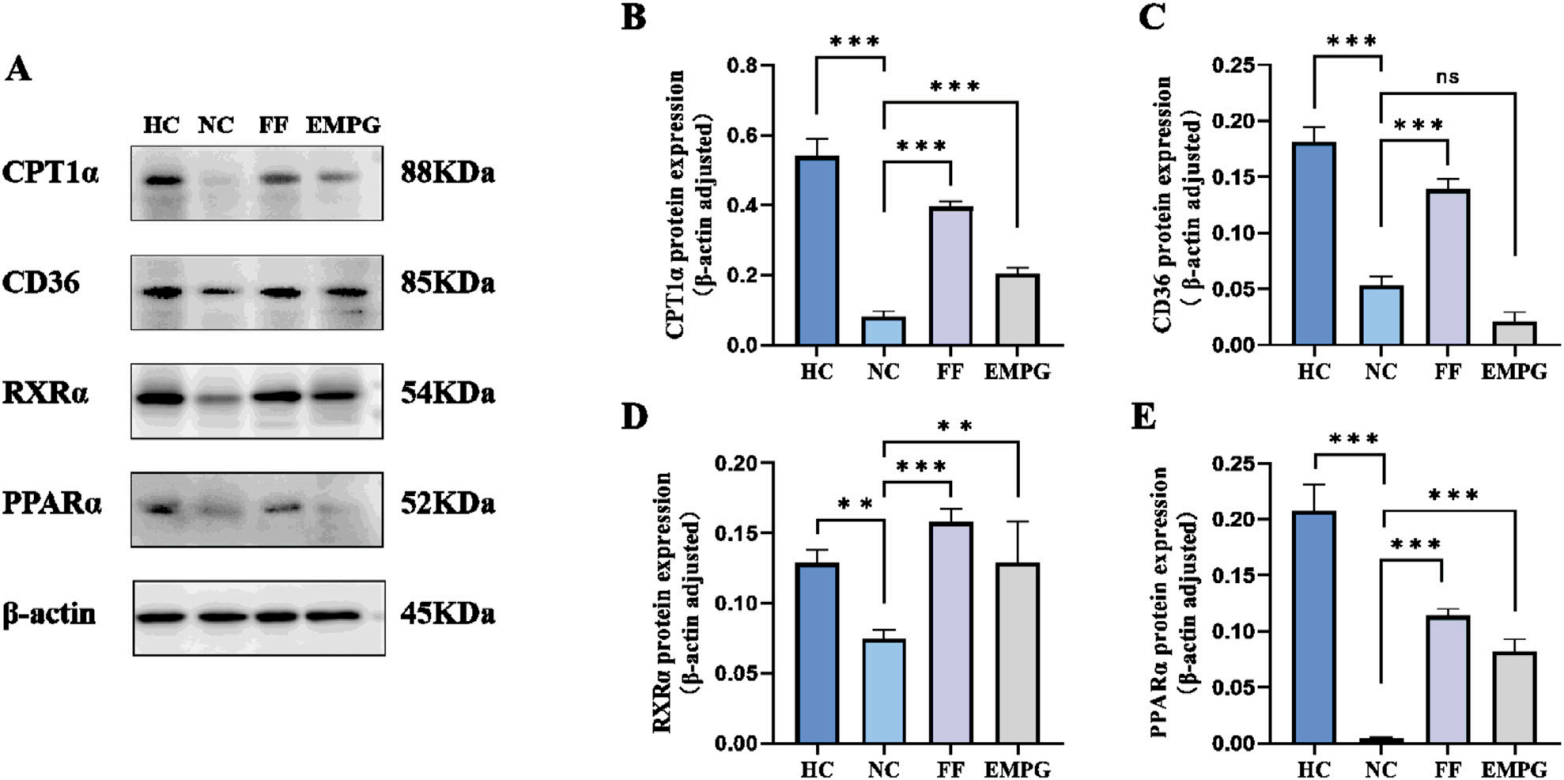
Changes in Western blot results after 4 weeks of drug treatment in rats of each group. **(A)** Plot of the typical bands of the lipid metabolism pathway proteins CPT1α, CD36, RXRα, and PPARα. **(B–E)** Quantitative analysis of PPARα, RXRα, CPT1α, and CD36 protein expression status. *For ns, P > 0.05,* ^**^ *P < 0.01, and* ^***^ *P < 0.001.*

**FIGURE 7:**
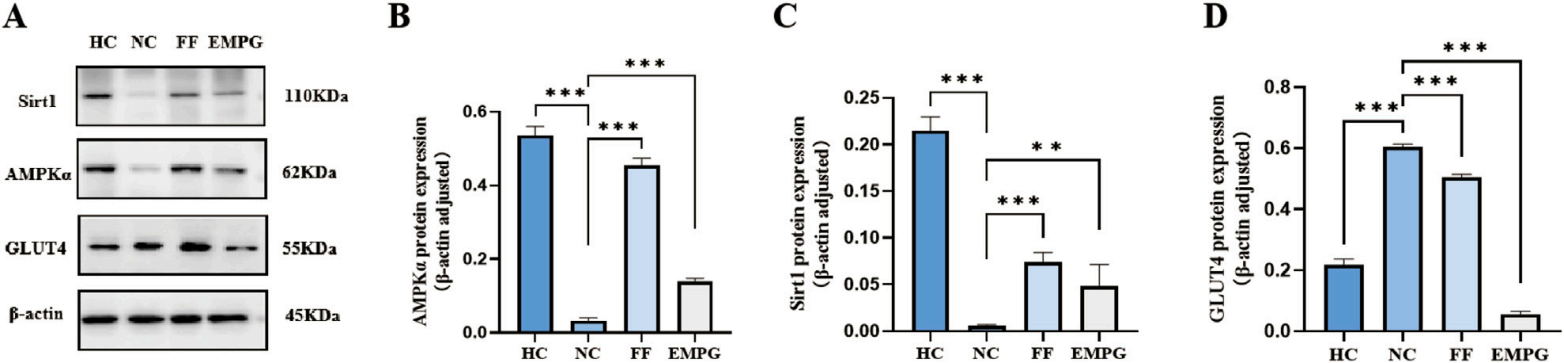
Changes in Western blot results after 4 weeks of drug treatment in rats of each group. **(A)** Plot of the typical bands of the energy homeostasis proteins Sirt1 and AMPKα and the glucose metabolism pathway protein GLUT4. **(B-D)** Quantitative analysis of the protein expressions of Sirt 1, AMPKα, and GLUT 4. **P < 0.01 and ***P < 0.001.

According to the Western blot results of Figures 6, 7, we can observe that, after 4 weeks of drug treatment, with regard to the lipid metabolism pathway proteins PPARα, RXRα, CPT1α, and CD36, protein expression was lower in the NC group than in the HC group, and the difference was statistically significant (p < 0.001, p < 0.01, p < 0.001, and p < 0.001, respectively); protein expression in the FF group was significantly increased compared to that in the NC group, and the difference was statistically significant (all were p < 0.001); protein expressions of the EMPG group, except for the CD36 (p > 0.05), PPARα, RXRα, and CPT1α, were significantly increased compared to that in the NC group, and the differences were statistically significant (p < 0.001, p < 0.01, and p < 0.001, respectively). Second, regarding the energy steady-state proteins Sirt1 and AMPKα, the protein expression of the HC group was significantly higher than that of the NC group, and the difference was statistically significant (p < 0.001); the FF group showed a significant increase, and the difference was statistically significant (both p < 0.001); the protein expression in the EMPG group showed a significant increase compared with that in the NC group, and the difference was statistically significant (p < 0.001 and p < 0.01, respectively). Finally, for the glycolytic pathway protein GLUT4, the protein expression in the HC group was significantly lower than that in the NC group, and the difference was statistically significant (p < 0.001); the FF group’s expression was slightly lower than that of the NC group (p < 0.001); the protein expression was higher than in the NC group (P < 0.001).

In conclusion, the cardiac energy metabolism level of HF rats is significantly lower than that of normal healthy rats. FF treatment can increase the protein expression of the lipid metabolism pathway and restore the energy supply level of the heart to some extent; EMPG can not only increase the protein expression of the lipid metabolism pathway but also inhibit the protein expression of the glycolysis pathway and glycolysis, thus improving the energy supply level of the heart to improve the effect of HF.

### 3.5 The corresponding gene expression of the glucose and lipid metabolism pathway proteins

**FIGURE 8:**
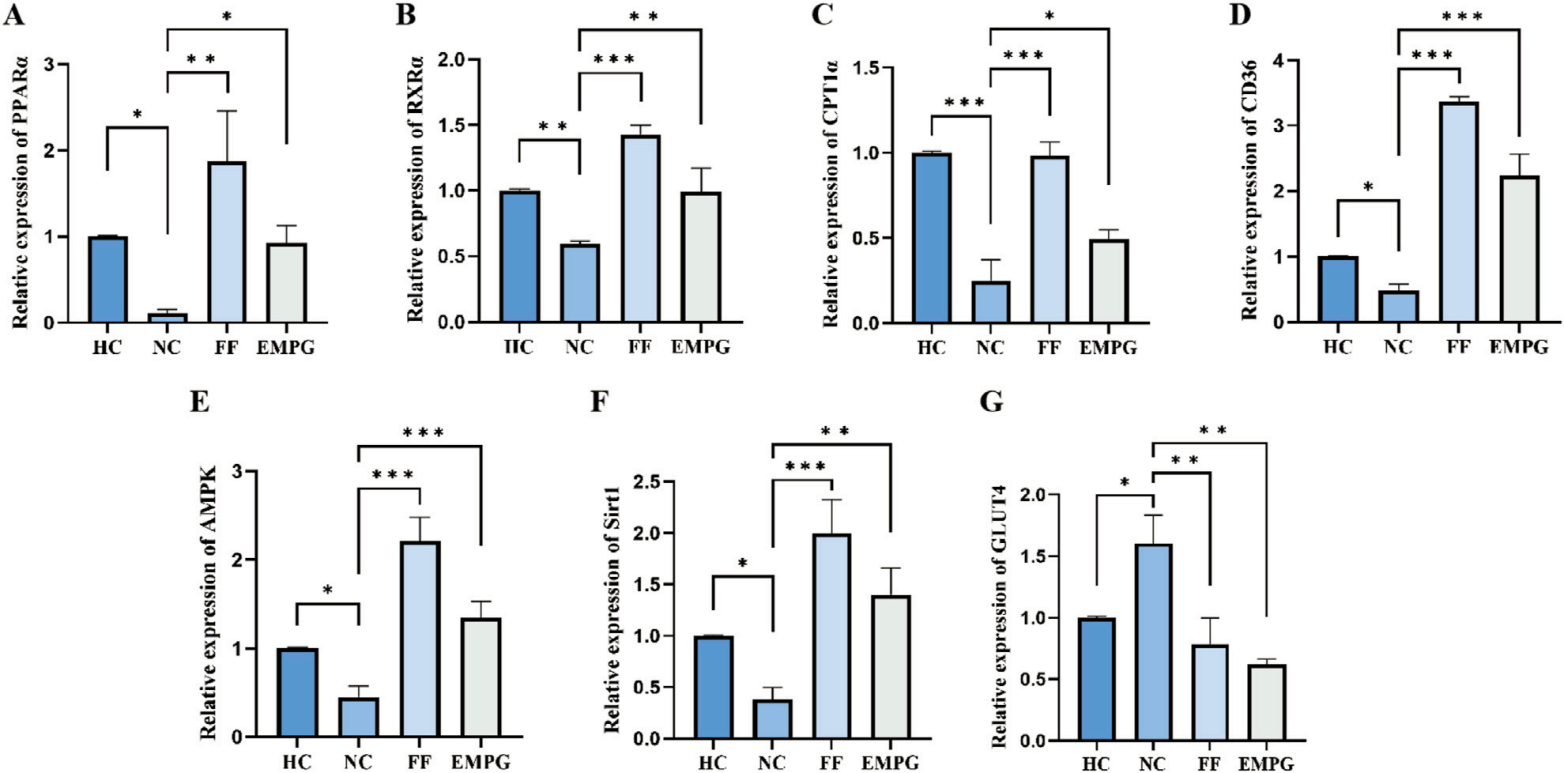
Changes in RT-PCR results after 4 weeks of drug treatment in rats in each group. **(A-G)** Relative expression of PPARα, RXRα, CPT1α, CD36, AMPKα, Sirt1, and GLUT4 genes. *P > 0.05,* ^*^ *P < 0.05,* ^**^ *P < 0.01,* and ^***^ *P < 0.001.*

The RT-PCR results shown in Figure 8 show that after 4 weeks of treatment with the drug, regarding the gene expression corresponding to the lipid metabolism pathway proteins PPARα, RXRα, CPT1α, and CD36, the HC group showed a significantly higher expression than the NC group, and the differences were statistically significant (p < 0.05, p < 0.01, p < 0.001, and p < 0.05, respectively). The expression of genes corresponding to lipid metabolism proteins in the FF group was significantly improved compared with that of the NC group, and the differences were statistically significant (p < 0.01, p < 0.001, p < 0.001, and p < 0.001, respectively). The expression of the genes corresponding to the metabolic pathway proteins in the EMPG group has a significantly improved performance compared to that in the NC group, and the differences were statistically significant (p < 0.05, p < 0.01, p < 0.05, and p < 0.001, respectively). Second, regarding the gene expression corresponding to the energy steady-state proteins Sirt1 and AMPKα, the HC group’s expression was significantly higher than that of the NC group, and the difference was significant (both p < 0.05); the FF group showed a significant increase, and it was statistically significant (both p < 0.001); the EMPG group showed a significant increase compared to the NC group, and the differences were significant (p < 0.001 and p < 0.01, respectively). Finally, regarding the expression of the gene corresponding to the glycolytic pathway protein GLUT4 was significantly lower in the HC group than in the NC group, which was statistically significant (p < 0.05); the FF group showed a statistically lower difference compared to the NC group (p < 0.01); the protein expression was compared to that in the NC group, and the difference was statistically significant (P < 0.01).

In conclusion, the cardiac energy metabolism level of HF rats is significantly lower than that of normal healthy rats. FF treatment can increase the expression of genes corresponding to the lipid metabolism pathway proteins and restore the energy supply level of the heart to some extent; EMPG can not only increase the protein expression of the lipid metabolism pathway proteins but also inhibit the expressions of genes corresponding to the glycolytic pathway proteins and inhibit the glycolysis level, thus improving the overall energy supply level of the heart to improve the effect of HF.

## 4 Discussion

This study, utilizing ^18^F-FDG MicroPET/CT dynamic imaging combined with molecular biology techniques, systematically reveals, for the first time, the dual mechanism by which the SGLT2 inhibitor EMPG improves energy metabolism disorders in HF by regulating the PPARα signaling pathway. We found that EMPG not only mimics the effects of the PPARα agonist FF by significantly upregulating key fatty acid oxidation proteins (PPARα, RXRα, and CPT1α) and energy sensors (AMPKα and Sirt1) to promote lipid metabolism but also uniquely suppresses glucose transporter GLUT4 expression and MRGlu, thus reversing the pathological shift of myocardial metabolic substrate toward glycolysis in the HF context. This “lipid-promoting, glucose-suppressing” metabolic remodeling ultimately improves myocardial fibrosis. To elucidate the cardioprotective effects of SGLT2 inhibitors, we will sequentially discuss their mechanisms based on the findings from this experiment.

### 4.1 ^18^F-FDG PET/CT and glucose metabolism rate

In the normal physiological state, the heart mainly provides energy through fatty acid β oxidation (60%–90%), followed by glucose (10%–30%) and ketone body (5%); after the HF development stage, the proportion of fatty acid oxidation decreases, and glucose glycolysis and ketone body metabolism gradually become the main modes for energy supply. The decrease in fatty acid oxidation cannot be completely compensated by the increase in glycolysis, and the accumulation of lactic acid, reactive oxygen and excess ketone body, and harmful lipids will further aggravate HF. This is the mechanism of the change in myocardial energy metabolism during the progression of HF, and the change in the extent of fatty acid β oxidation, glucose glycolysis, and ketone body utilization occupies an important position (Saucedo-Orozco et al., 2022). In the study by Yang et al. (2021) in normal rats and in rats with a canagliflozin diet, on comparing the changes in different environmental respiratory quotient combined with ^18^F-FDG PET/CT, it was found that canagliflozin could increase fat sympathetic innervation and fat mobilization. Cinti et al. (2023), in the study of patients with type 2 diabetes and patients with stable coronary heart disease, compared the sub-epicardial fat thickness and the degree of myocardial glucose uptake in the patients by ^18^F-FDG PET/CT and found that dapagliflozin can reduce epicardial thickness and myocardial glucose uptake in patients with type 2 diabetes and stable coronary heart disease. It is consistent with the previous result that ^18^F-FDG PET/CT imaging gradually decreases cardiac glucose uptake after EMPG treatment (Figure 2, Figure B). Unlike the previous study, MRGlu can further quantify the change in myocardial glucose uptake in ^18^F-FDG PET/CT images of HF rats, which more intuitively reflects the changes in myocardial glucose metabolism level in HF rats.

### 4.2 SGLT2 inhibitors and PPARα agonist

Previous studies have found that the PPAR family is divided into three functionally distinct subtypes, among which PPARα is highly expressed in myocardial tissue, which can promote FAO expression, and it is an important control factor of fatty acid oxidation in the heart (Montaigne et al., 2021; Drosatos et al., 2016; Prosdocimo et al., 2015). Kaimoto et al. (2017) analyzed PPARα knockout rats for cardiac function changes and found that PPARα can maintain myocardial energy supply by regulating FAO expression. Wei et al. (2020) studied the effects of canagliflozin drug and normal diet with high-fat diet rats and found that canagliflozin could potentially upregulate the expression of the PPARα series of signaling pathway proteins to improve the effect of glucose–lipid metabolism induced by a high-fat diet. Liu et al. (2016), in a study of FF intervention in rabbits with atrial fibrillation, found that FF can enhance the expressions of PPARα and Sirt1 and improved atrial metabolism during AF. Consistent with the results of WB and RT-PCR in the present study, the HF rats showed a significantly decrease in the expressions of PPARα, RXRα, CPT1α, and CD36 when compared with the healthy rats. After treatment with EMPG and FF, the results also showed an increased expression of PPARα, RXRα, and CPT1α, and PPARα activation is similar to that with EMPG and FF; in contrast, EMPG showed an inhibition of CD36 expression; this is similar to the findings of Aragón-Herrera et al. (2019), who found that EMPG can significantly reduce CD36 expression, thus reducing the amount of toxic lipids in the heart and improving the heart function.

Santos-Gallego et al. (2019) induced HF by performing stenosis of the proximal left anterior descending artery in non-diabetic pigs, and the pigs were treated with EMPG during February. After evaluation by cardiac magnetic resonance imaging and three-dimensional echocardiography and analyzing the consumption of myocardial metabolites, taking myocardial samples for molecular evaluation, it was concluded that EMPG can shift myocardial energy metabolism from glucose to ketone body and free fatty acid substrate of lipid metabolism through activation of enhanced AMPK and PGC1α signaling, and it can enhance the LV systolic function and improve the LV remodeling. Cai et al. (2024), through a meta-analysis, series of imaging data, and histological pathology analysis of EMPG drugs in diabetic mice, found that EMPG regulated ketone body metabolism and oxidative stress, improving the mitochondrial dysfunction in patients with diabetic cardiomyopathy, thus improving its energy supply level. Kolb et al. (2021) found that SGLT2 inhibitors can increase the level of ketone body metabolism to activate the master regulator of the cytoprotective mechanism, the nuclear factor erythroid-associated factor 2 (Nrf 2), Sirt, and AMPK, to upregulate the cellular antioxidant levels and anti-inflammatory activity. Thus, improving mitochondrial function and growth, DNA repair, and autophagy contribute to its cardioprotective function. Yang et al. (2020) studied the stromal vascular components (SVFs) from subcutaneous adipose tissue in mice treated with canagliflozin to obtain their oxygen consumption rate and PCR analysis and concluded that canagliflozin could increase the phosphorylation of AMPK and the expressions of Sirt 1 and PGC-1α to improve the mitochondrial remodeling and improve the functional efficiency. Koyani et al. (2020) used lipopolysaccharide to induce inflammatory responses in cardiomyocytes and macrophages *in vitro* and *in vivo*, and the analysis of their intracellular signaling pathways showed that SGLT2 inhibitors can increase the expression of anti-inflammatory M2 marker proteins in macrophages to enhance the anti-inflammatory effect and can activate AMPK signaling proteins to prevent lipopolysaccharide-induced ATP/ADP depletion to achieve the protective cardiac benefits of reducing energy expenditure. Costantino et al. (2023) compared metabolic heart disease model mice with normal mice; after 4 weeks of treatment by intraperitoneal Sirt1 supplementation, they found that the reduced cardiac expression level of Sirt1 was correlated with increased gene expressions associated to triacylglycerol and PPAR in the myocardium. Many of the aforementioned studies, with different methods, have proved that SGLT2 inhibitors can affect the cardiac energy metabolism substrate changes to increase the expressions of AMPK and Sirt1 and improve energy supply efficiency to improve heart function, and the experimental results of HF rats by EMPG treatment before and after the expression of AMPK, with increased Sirt1, further verified that EMPG can not only improve the cardiac energy efficiency of DM rats but also showed the same effect in HF rats.

In addition, the results of this study showed that EMPG inhibited GLUT4 expression. Considering the above discussion, we can prove that EMPG activated PPARα, RXRα, and CPT1α similar to FF and inhibited the expression of GLUT4, promoting lipid metabolism, inhibiting glycolysis, improving the cardiac energy efficiency, inhibiting CD36 expression, and jointly improving cardiac function.

### 4.3 Heart pathology

Finally, regarding the change in pathological slices before and after the treatment in this study, only the EMPG group showed the recovery of myocardial arrangement and decreased myocardial fibrosis after treatment, combined with the decrease in GLUT4 protein expression in the WB results. The increase in GLUT4 caused by external factors was found by Faramoushi et al. (2016) aggravated myocardial glucose homeostasis and myocardial structural damage, and it once again verified that EMPG reversed the effects of ventricular remodeling in HF rats, and this effect was achieved by adjusting the GLUT4 protein levels.

In conclusion, in the development of HF, energy metabolism occurs, and the use of EMPG can change this process, improve the level of cardiac energy metabolism, and promote the reversal of cardiac remodeling. Meanwhile, the treatment of HF with EMPG is accompanied by the increase in PPARα, RXRα, and CPT1α metabolic pathway proteins, and this process can be visualized by ^18^F-FDG PET/CT imaging.

## 5 Conclusion

MRGlu obtained by ^18^F-FDG MicroPET/CT imaging can show that increased glucose metabolism appears in rats at the early stage of HF. In addition, after 4 weeks of EMPG treatment, significantly decreased glucose metabolism in the heart of rats with chronic HF can be observed by HE and MASSON staining. EMPG has a certain effect on improving cardiac fibrosis and promoting the reversal of ventricular remodeling; detection of WB and PCR of PPARα-related signaling and GLUT4 glucose metabolism pathway in myocardial tissues of rats with HF confirms that the expression of lipid metabolism signaling protein decreases, and glucose metabolism signaling protein expression is impaired in the early stage of HF. However, EMPG could upregulate the expressions of PPARα, RXRα, and CPT1α lipid metabolism signaling and AMPKα and Sirt1 proteins, downregulate GLUT4 glucose metabolism signaling protein signaling pathway protein expression, and downregulate the expression of CD36 to achieve the improvement of heart lipid metabolism, reduce heart sugar metabolism, and enhance the efficiency of heart energy supply to improve the purpose of heart energy metabolism in chronic HF.

## 6 Limitations

There may be some possible limitations in this study. First, being limited by the acquisition conditions, the rat cardiac glucose metabolism rate needs to be obtained by dynamic 1 h continuous acquisition; therefore, the data amount of MRGlu is less. Second, we studied the effect of EMPG for only 4 weeks and observed the role of EMPG in improving energy metabolism dysfunction in HF; however, chronic HF treatment is a long-term process, and whether the use of EMPG changes the indicators of LV function and its long-term therapeutic effect still needs to be further explored. Finally, this study was originally planned to evaluate the myocardial fatty acid metabolism by ^18^F-FTHA PET/CT. Limited by the preparation of imaging agents and the failure to demonstrate complementation with ^18^F-FDG PET/CT imaging, we proceed to ^18^F-FTHA PET/CT imaging compared with ^18^F-FDG PET/CT imaging to further clarify the level of cardiac energy supply altered by the substrate proportion changes in cardiac energy metabolism.

## Data Availability

The data cohorts used in this manuscript are available from the corresponding author on reasonable request.

## Abbreviations

HF, heart failure; PPARα, peroxisome proliferator-activated receptor alpha; RXRα:retinoic X receptor α; CPT1α, carnitine palmitoyltransferase 1α; CD36, platelet glycoprotein 4; AMPKα, adenosine 5‘-monophosphate (AMP)-activated protein kinase α; Sirt1, silent information regulator 1; SGLT2, sodium-dependent glucose transporters 2; MRGlu, myocardial glucose metabolism rate; EMPG, empagliflozin; FF, fenofibrate; RT-PCR, real-time quantitative amplification analysis; FAO, fatty acid oxidation enzymes; β-OHB, β-hydroxybutyrate; FFA, free fatty acids; PET/CT, positron emission tomography/computed tomography; HE, hematoxylin and eosin staining; Nrf 2, erythroid-associated factor 2; SVFs, stromal vascular components; PGC1, peroxisome proliferator-activated receptor γ coactivator l; FTHA, fluoro-6-thia-heptadecanoic acid.
