# Supplementary material for: Empagliflozin’s cardioenergetic protective effects through PPARα pathway modulation in heart failure
Source: Front Pharmacol. 2025 Oct 17;16:1636810. doi: 10.3389/fphar.2025.1636810 (PMC12575102; doi:10.3389/fphar.2025.1636810)

Below are 14 samples (left to right) in the following order:

1HC, 2NC, 3FF, 4FF, 5EMPG, 6EMPG, 7HC, 8NC, 9FF, 10EMPG, 11HC, 12NC, 13FF, 14EMPG.

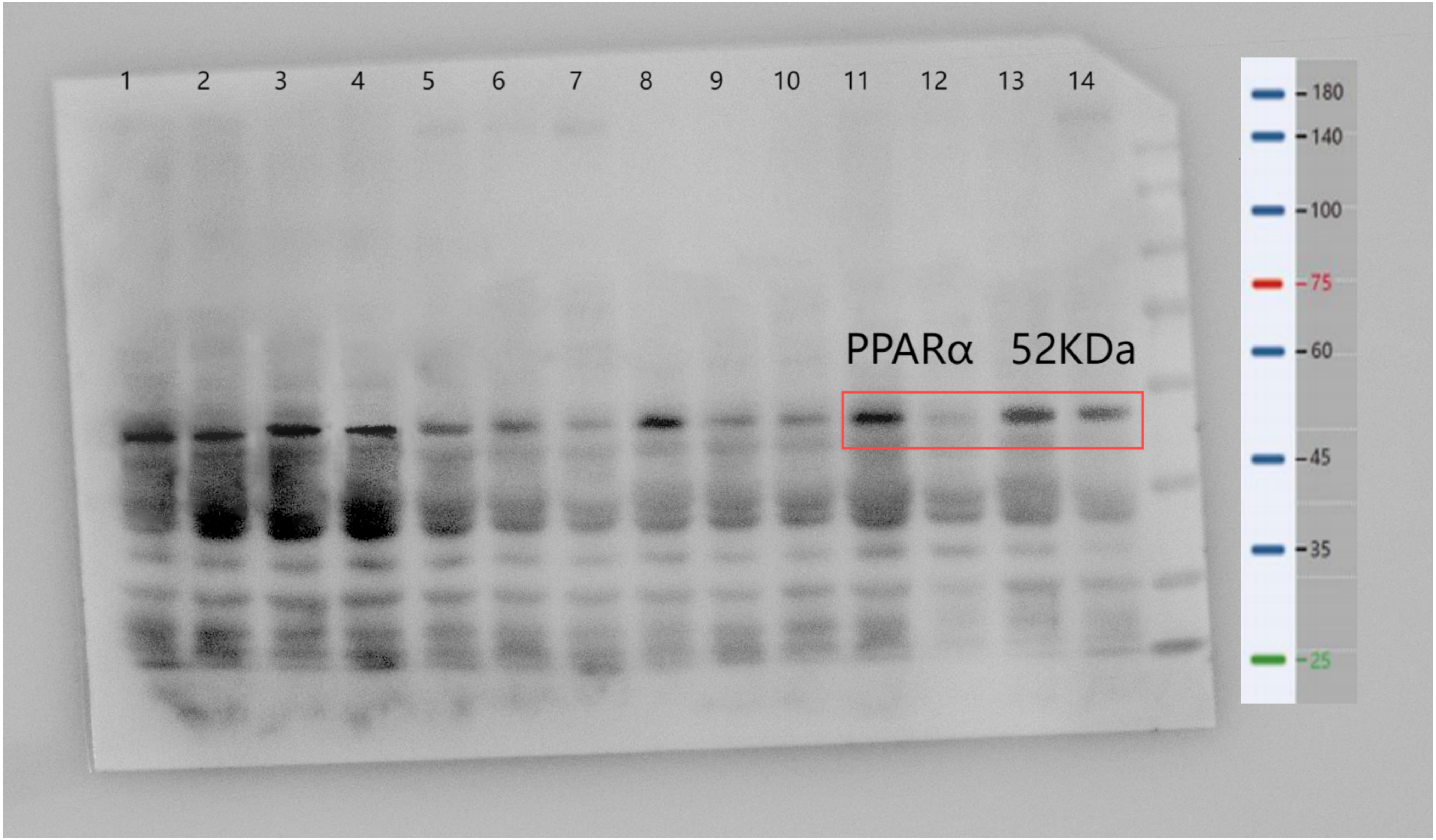

Below are 14 samples (left to right) in the following order:

1HC, 2NC, 3FF, 4FF, 5EMPG, 6EMPG, 7HC, 8NC, 9FF, 10EMPG, 11HC, 12NC, 13FF, 14EMPG.

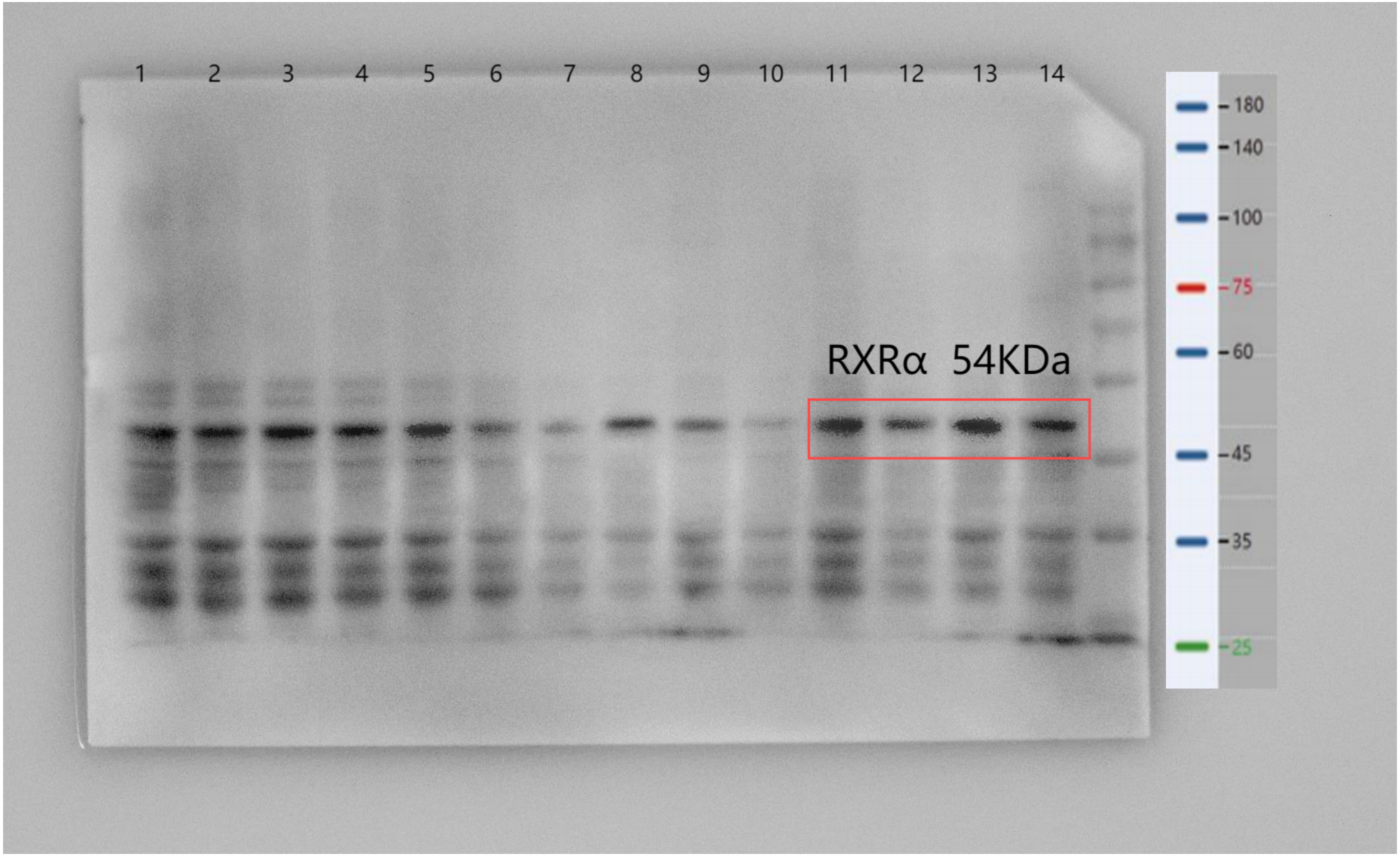

Below are 14 samples (left to right) in the following order:

1HC, 2NC, 3FF, 4FF, 5EMPG, 6EMPG, 7HC, 8NC, 9FF, 10EMPG, 11HC, 12NC, 13FF, 14EMPG.

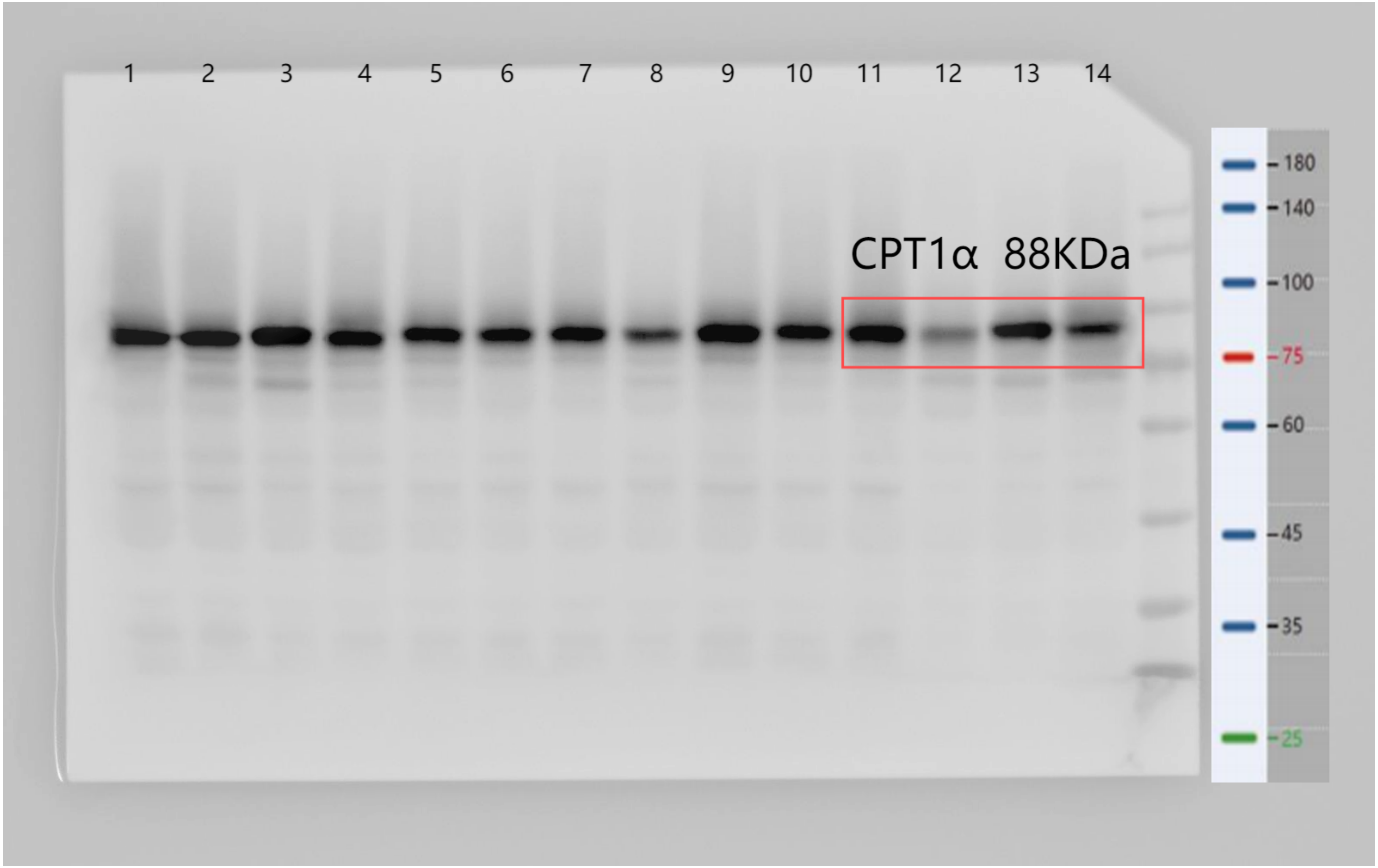

Below are 14 samples (left to right) in the following order:

1HC, 2NC, 3FF, 4FF, 5EMPG, 6EMPG, 7HC, 8NC, 9FF, 10EMPG, 11HC, 12NC, 13FF, 14EMPG.

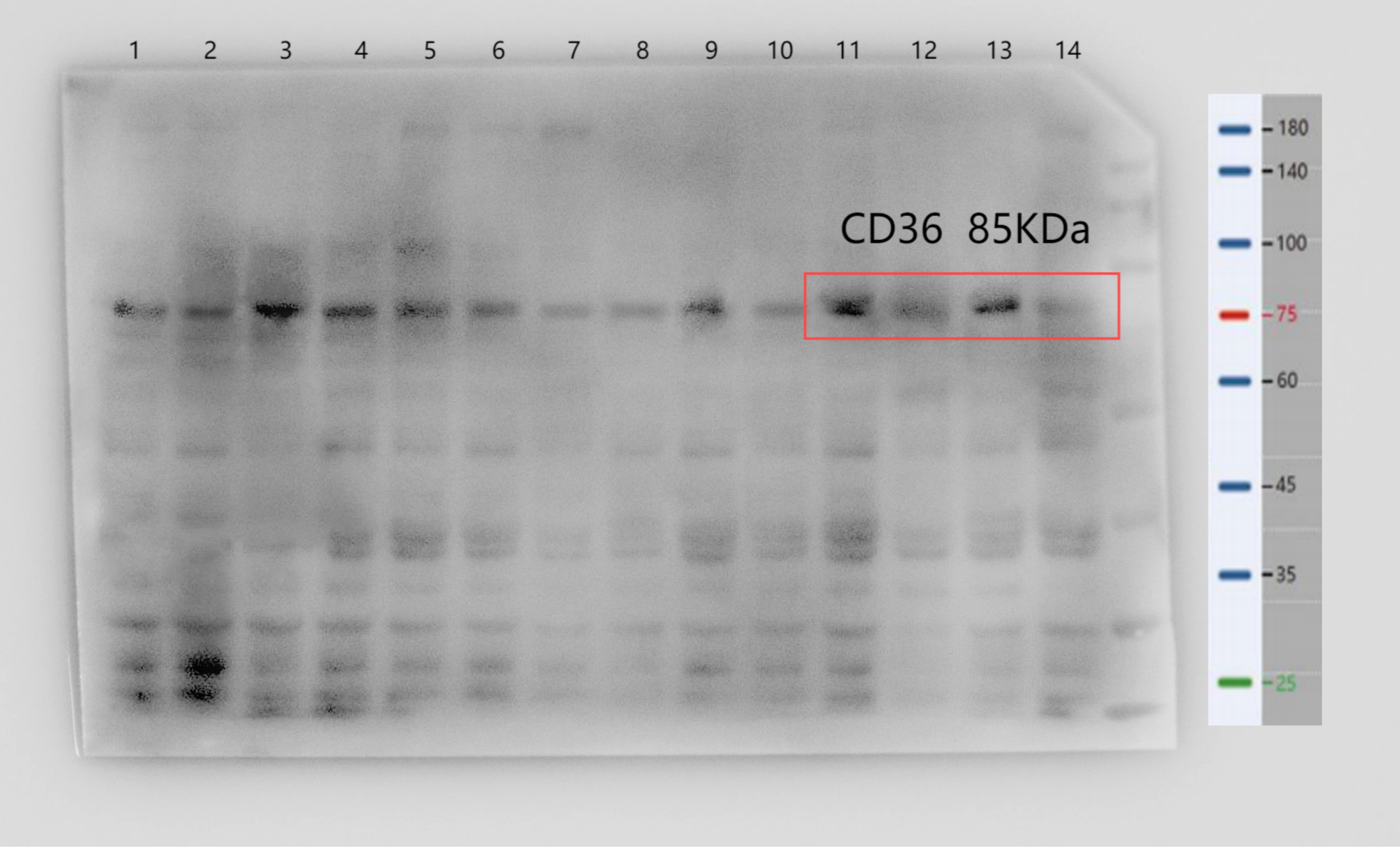

Below are 14 samples (left to right) in the following order:

1HC, 2NC, 3FF, 4FF, 5EMPG, 6EMPG, 7HC, 8NC, 9FF, 10EMPG, 11HC, 12NC, 13FF, 14EMPG.

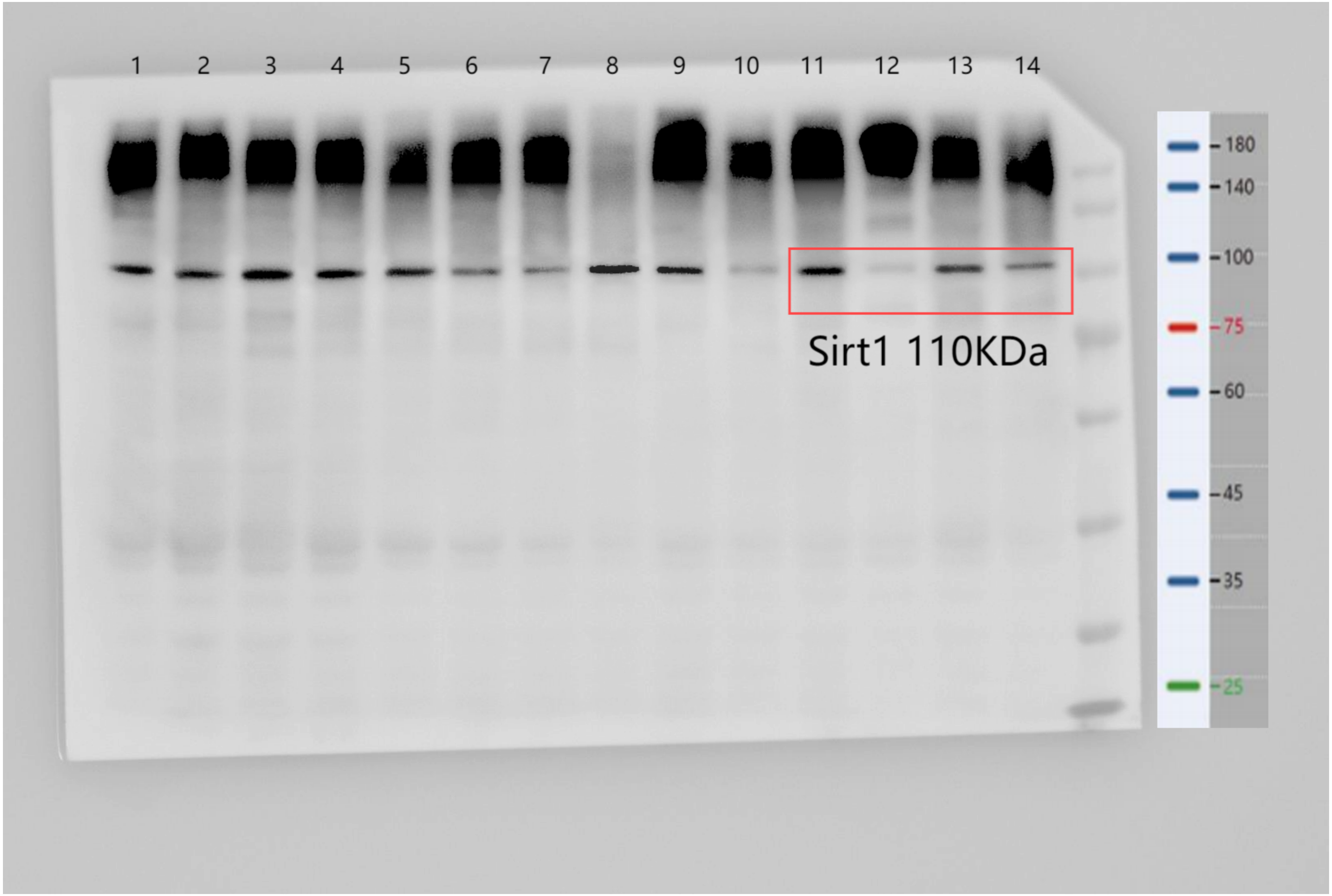

Below are 14 samples (left to right) in the following order:

1HC, 2NC, 3FF, 4FF, 5EMPG, 6EMPG, 7HC, 8NC, 9FF, 10EMPG, 11HC, 12NC, 13FF, 14EMPG.

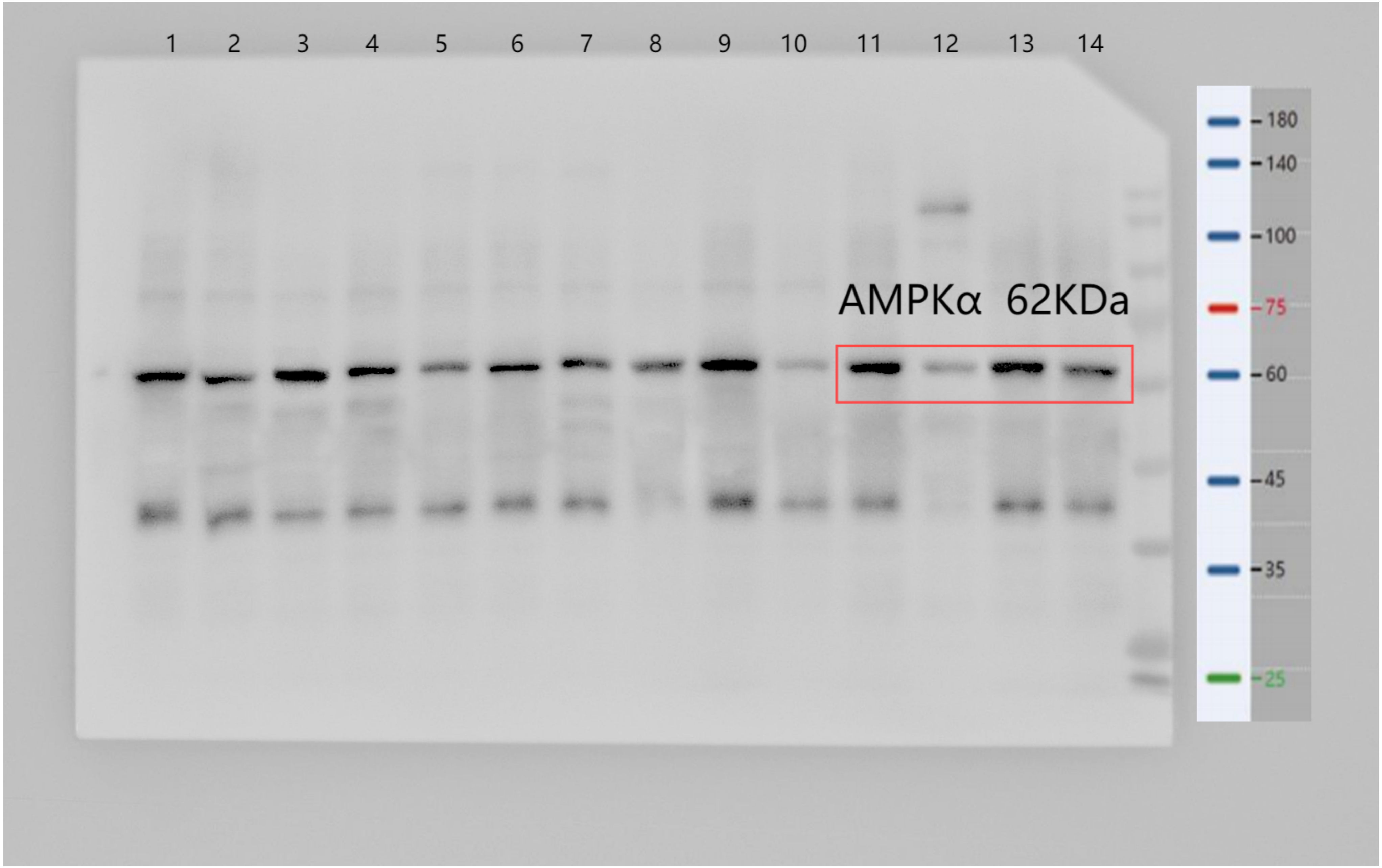

Below are 14 samples (left to right) in the following order:

1HC, 2NC, 3FF, 4FF, 5EMPG, 6EMPG, 7HC, 8NC, 9FF, 10EMPG, 11HC, 12NC, 13FF, 14EMPG.

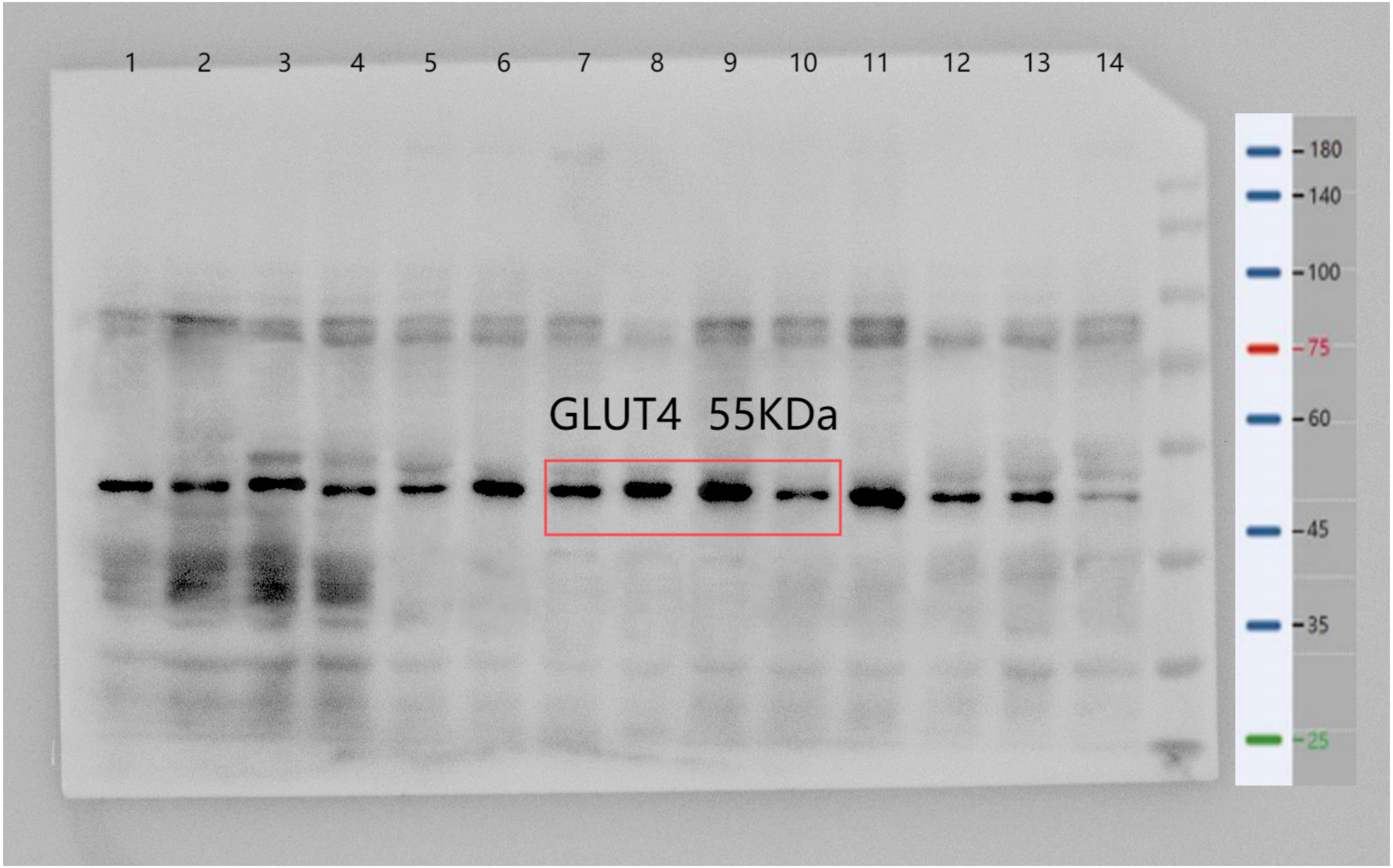

Below are 14 samples (left to right) in the following order:

1HC, 2NC, 3FF, 4FF, 5EMPG, 6EMPG, 7HC, 8NC, 9FF, 10EMPG, 11HC, 12NC, 13FF, 14EMPG.

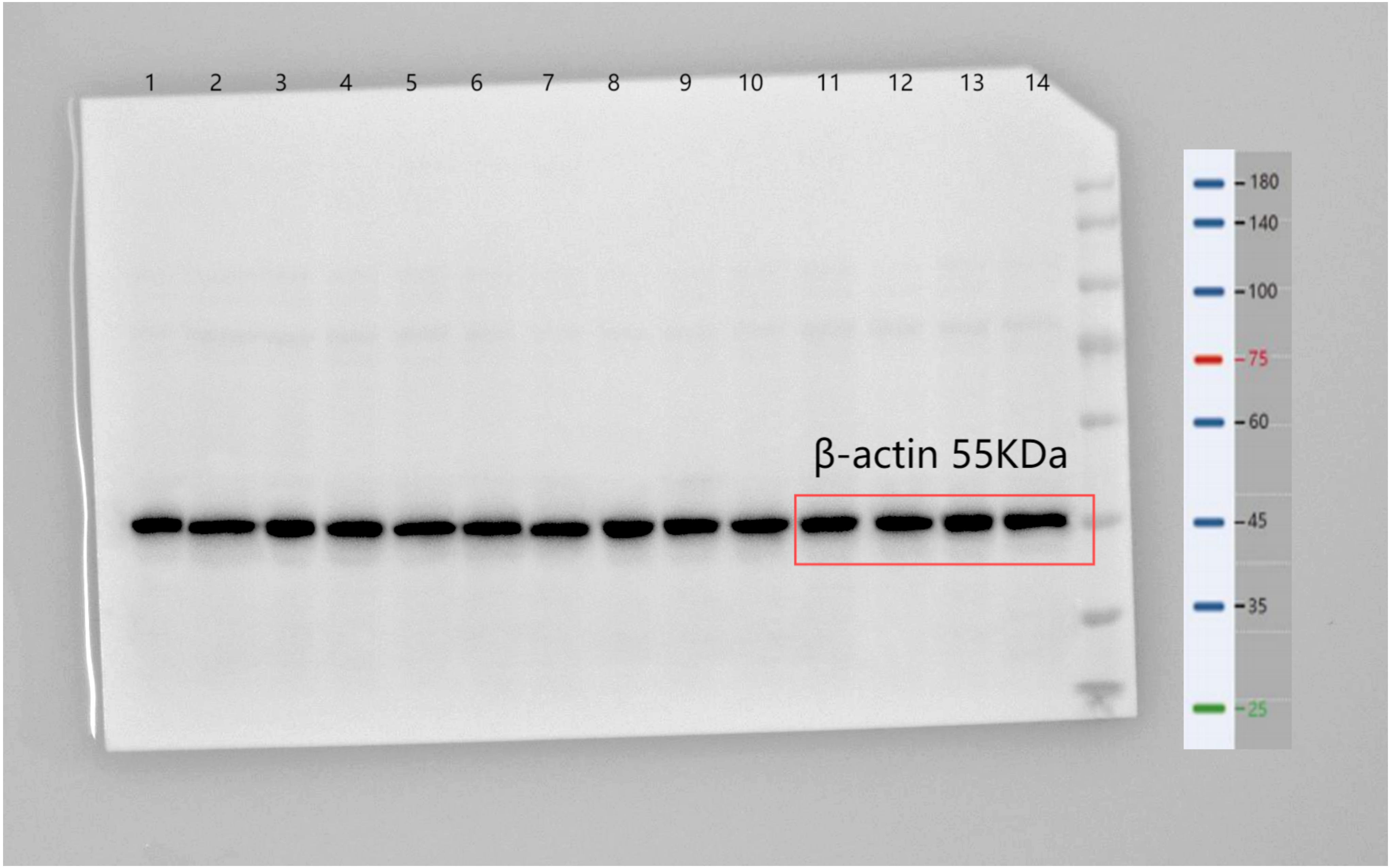

Supplement: Supplementary file 1 [file DataSheet1.pdf]
